# Novel Approach to Automated Flow Titration for the Determination of Fe(III)

**DOI:** 10.3390/molecules25071533

**Published:** 2020-03-27

**Authors:** Joanna Kozak, Justyna Paluch, Marek Kozak, Marta Duracz, Marcin Wieczorek, Paweł Kościelniak

**Affiliations:** 1 Faculty of Chemistry, Jagiellonian University, Gronostajowa 2, 30-387 Krakow, Poland; 2 Oil and Gas Institute—National Research Institute, Lubicz 25A, 31-503 Krakow, Poland

**Keywords:** titration, flow analysis, Fe(III), Fe(II) determination, speciation analysis

## Abstract

A novel approach to automated flow titration with spectrophotometric detection for the determination of Fe(III) is presented. The approach is based on the possibility of strict and simultaneous control of the flow rates of sample and titrant streams over time. It consists of creating different but precisely defined concentration gradients of titrant and analyte in each successively formed monosegments, and is based on using the calculated titrant dilution factor. The procedure was verified by complexometric titration of Fe(III) in the form of a complex with sulfosalicylic acid, using EDTA as a titrant. Fe(III) and Fe(II) (after oxidation to Fe(III) with the use of H_2_O_2_) were determined with good precision (CV lower than 1.7%, *n* = 6) and accuracy (|RE| lower than 3.3%). The approach was applied to determine Fe(III) and Fe(II) in artesian water samples. Results of determinations were consistent with values obtained using the ICP–OES reference method. Using the procedure, it was possible to perform titration in 6 min for a wide range of analyte concentrations, using 2.4 mL of both sample and titrant.

## 1. Introduction

Techniques of flow analysis offer many possibilities of automation of analytical methods, including titration analysis. Using flow-based systems, titration has been performed based on the conventional procedure [1,2,3,4,5,6,7], and many novel approaches have also been developed for its implementation [8,9,10,11,12,13,14,15,16,17,18,19,20,21,22,23,24,25,26,27,28,29,30,31,32,33,34,35]. They differ in terms of the way of introducing the sample into the flow system, merging it with the titrant solution to create an analyte concentration gradient, and, consequently, the way of determining the analyte concentration. Generally, they can be divided [8] into titration based on the flow of a sample stream or segment, formation of a sample monosegment, or a combination of different ways of conducting titration in flow mode.

Titration based on continuous flow is generally performed in two modes. The first is based on changing the flow rate of only one stream (usually titrant [9,10,11]) and creating a single analyte concentration gradient using, e.g., a peristaltic pump. In this way, the analyte concentration was determined based on analytical calibration (a set of standard solutions was subjected to the titration procedure in the same instrumental conditions as the sample) [9,10] or on the relationship between the time necessary to achieve the endpoint of titration and the flow rate of the titrant (introduced using a double-plunger micropump) measured at the endpoint of titration [11]. The other approach is based on changing the flow rates of the sample and titrant streams while keeping the sum of the flow rates constant [12,13,14,15,16,17,18,19,20,21,22]. In this (latter) case, a single concentration gradient with analytical calibration [12] or a triangle-programmed concentration gradient of the analyte was formed [12,13,14,15,16,17]. To this end, e.g., two streams of titrant and analyte were introduced into the system using peristaltic pumps, first increasing the titrant flow rate from zero to a value corresponding to zero sample flow rate and then, at the same rate, reducing to zero again. As a result, two titration curves were obtained and the time corresponding to the difference between the endpoints of titration detected on the curves was calculated [12]. Triangle-programmed flow titration with linear concentration gradients was also performed using computer-controlled micropumps. Under defined conditions, it was possible to perform determinations without analytical calibration [13]. In flow titration, called continuous feedback-based titration [14,15,16], at a constant sum of the flow rates of the titrant and sample, the linear change of titrant flow rate was related to the controller output voltage dependency. During titration, the voltage increased until a certain value, U_H_, was reached, at which the time corresponding to the endpoint of the titration was exceeded. Then, the voltage decreased in the same range until the U_L_ value was reached. The controller output voltage at the endpoint of titration was the arithmetic mean of the U_H_ and U_L_ values. The method enabled the determination of analyte concentration using an appropriate formula [14,16] or analytical calibration [15,16]. Coulometric titration with a continuous sample flow in which the concentration gradient of the analyte was associated with the magnitude of the rectangular [17] or triangular [18,19,20] current pulse generating the titrant in a flow-through electrolytic vessel was also proposed.

Monosegmented flow titration is characterized by the formation of segments of liquid—each segment consists of the sample, titrant, and possibly a diluent—separated on both sides from the carrier by a segment of air (or inert gas) [21,22,23,24,25,26,27,28]. The components of the created monosegment are mixed in a reaction coil. In order to facilitate homogenization, several smaller segments may be introduced alternately instead of a single segment of the sample and the titrant [21,23]. For a monosegment prepared in this way, usually, a peak with a plateau is registered. While maintaining the size of the entire monosegment, the described procedure is repeated, changing the volume of the titrant segment introduced into the monosegment [21,24] or selecting the volume of both the titrant and the sample [23,25]. In this method of titration, the endpoint is determined on the basis of titration curves representing the relation between signal and titrant volume [23] or time [24] or using an appropriate numerical algorithm [21,25]. Finally, the analyte concentration can be calculated. In the system described in [26], titration was performed in a monosegment containing the sample to which the titrant portion was dosed by a burette. The monosegment content was mixed in a reaction coil and directed to the detector. After the peak was recorded, the direction of the flow was changed in order to introduce another portion of the titrant into the monosegment and continue the procedure. Monosegmented titration was also carried out using Lab-on-Valve [27] or sequential injection [28] systems in which segments of air, sample, indicator, titrant, and then air were introduced into the holding coil in turn. The content of the monosegment was homogenized by changing the direction of flow, after which the second of the introduced air segments was removed to waste, and solutions were directed to the detection channel to measure the signal. Calibration was performed using a set of standard solutions.

There is a group of flow titration methods that can be considered as a combination of various approaches. Among them is multicommutated continuous flow titration [29,30,31,32,33,34], which can be classified as a method in which the sample is introduced both as a stream and as a segment. This titration, provided that the sum of the volumes of two successive segments remains constant, can be implemented, e.g., by introducing alternately larger segments of the titrant and smaller segments of the sample into the stream of the sample [29,30,31], larger segments of sample, and smaller segments of titrant into the titrant stream [32,33], or by using binary search concepts [34]. Based on the titration curve representing the relationship between the registered signal and the time of analysis [29,30,31,32] or the number of delivery cycles of the titrant and the sample [33], the titration time or the cycle number corresponding to the achievement of the endpoint of titration was determined. The concentration of the analyte in the sample was determined using the time corresponding to the endpoint of titration and the theoretical model describing the change in the analyte concentration during the delivery of the titrant and sample to the mixing chamber. The concentration of the analyte in the sample was also determined based on calibration using a set of standard methods.

A tracer-monitored flow titration with spectrophotometric detection was developed to omit the stage of analytical calibration [35]. The approach requires simultaneous monitoring of two absorbing species, the titration indicator and the dye tracer [36]. The tracer is applied to estimate the instant sample and titrant volumetric fractions without the need for volume, mass, or peak width measurements [36]. The method was implemented using a single flow system and applied to perform triangle-programmed titration, and to establish concentration gradients along the sample zone in the flow injection technique.

In the present work, a simple approach to the automation of titration with spectrophotometric detection based on merging (in a strictly controlled way) sample and titrant streams introduced continuously into the detection system in the form of monosegments is presented. It consists of creating in the flow system different, but precisely defined concentration gradients of titrant and analyte in each of successively formed monosegments and is based on using the calculated titrant dilution factor. The procedure was verified and applied to complexometric Fe(III) titration.

## 2. Results and Discussion

### 2.1. Flow System Developed for Titration

**Figure 1:**
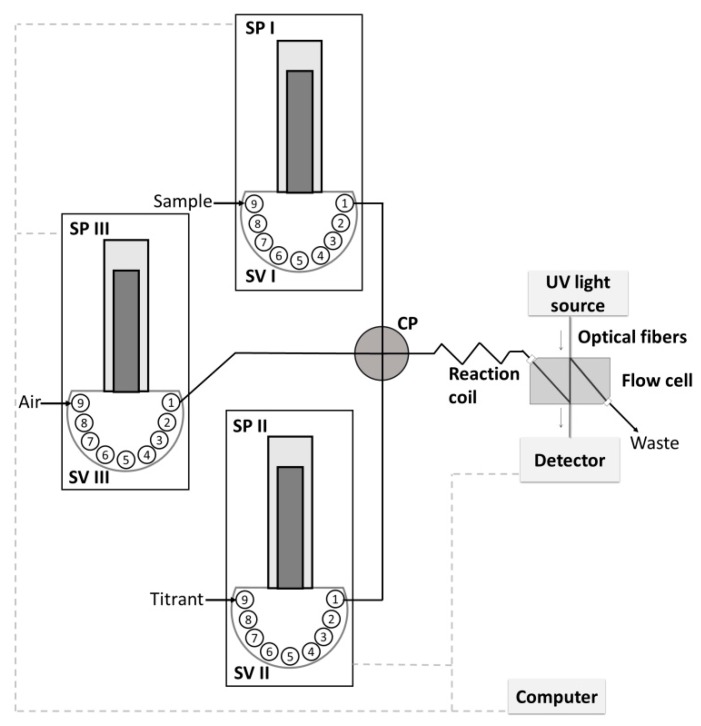
Flow system designed for the proposed titration procedure. SP—syringe pump, SV—selection valve, CP—confluence point.

The flow system proposed for titration is presented in Figure 1. It consists of three syringe pumps equipped with nine-position selection valves. Pump I and Pump II were used for sample and titrant propelling, respectively, whereas the third pump was used for air introduction to reduce the dispersion of solutions. Streams of sample and titrant solutions were introduced simultaneously into the system with established flow rates, joined at the confluence point, and merged in the mixing coil to complete the reaction. The product of the reaction was directed in a continuous way to the flow cell and appropriate signals were measured by the detector. In this way, the change in the titration curve was monitored continuously. Segments of air were introduced at strictly defined, subsequent stages of titration. Using the pumps, the flow rate of streams could be strictly defined and controllably changed in a discrete or continuous, and reproducible way. This became the basis for developing titration procedures based on using the calculated titrant dilution factor.

### 2.2. Procedure of Titration

Titration procedure consisted of creating different but precisely defined concentration gradients of titrant and analyte in each successively formed monosegments in order to facilitate the mixing of titrant and sample solutions, and in order to obtain monosegments containing precisely defined titrant (V_T_) and sample (V_S_) volumes. The procedure was carried out by simultaneously introducing into the monosegment the sample and titrant, each with a known, properly selected, flow rate. The first monosegment contained only the sample, and in the subsequent monosegments, the volume of sample was gradually decreased, whereas the volume of titrant increased by the same value (e.g., V_S_/V_T_ (µL): 300/0; 280/20; 260/40; 240/60; … 40/260; 20/280; 0/300) until the formation of a monosegment that contained only the titrant. In practice, the titration procedure was ended after obtaining the endpoint of titration. During the whole titration procedure, the total volume of the mixture (V_M_) in successive monosegments remained constant, whereas the titrant and analyte concentration increased and decreased linearly, respectively. Consequently, based on the volumes of appropriate solutions introduced with the use of syringe pumps, for each monosegment it was possible to calculate titrant dilution factor (f_T_) (Equation (1)):

f_T_ = V_T_/(V_T_ + V_S_) (1)

and sample dilution factor f_S_ (Equation (2)):

f_S_ = V_S_/(V_T_ + V_S_) (2)

**Figure 2:**
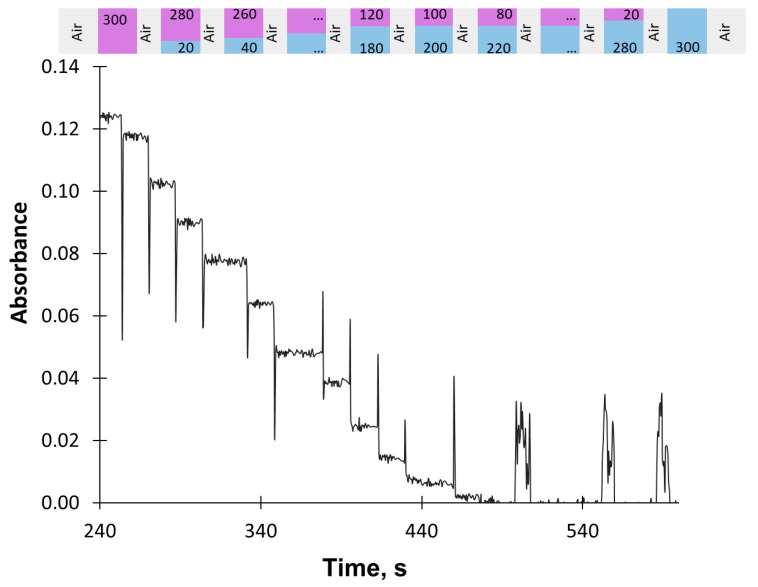
Composition of monosegments formed during the titration procedure (in µL) and signals registered during titration of Fe(III) (2.00 mg L^−1^) using EDTA (0.02 mmol L^−1^) as titrant.

**Figure 3:**
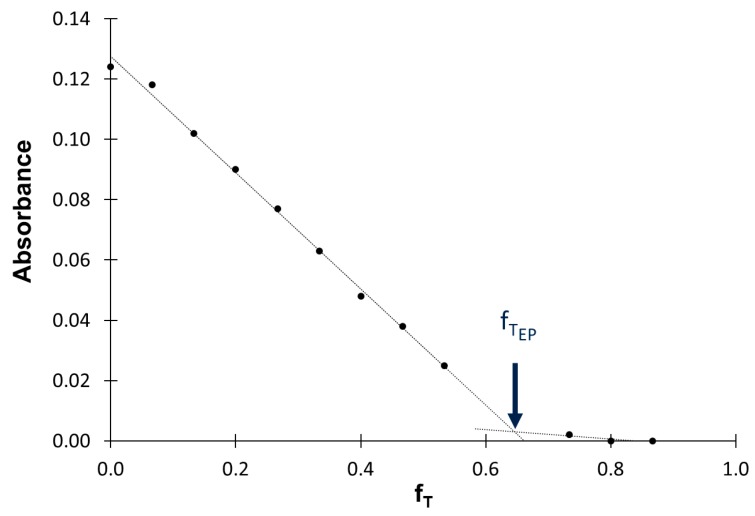
The way of determination of the endpoint of titration (EP) using titrant dilution factor f_T_ (details in the text).

It should be noted that, therefore, f_T_ = 1 − f_S_. The absorbance in the form of a steady signal was measured for each of the monosegments (Figure 2) and a titration curve showing the relationship between absorbance and the titrant dilution factor was prepared (Figure 3). Using this titration curve, the titrant dilution factor at the endpoint of titration f_TEP_ was determined. Analyte concentration (C_A_, g L^−1^) was calculated based on titrant concentration (C_T_, mol L^−1^) and titrant dilution factor determined at the endpoint of titration according to Equation (3):

C_A_ = (Q·C_T_·M_A_·f_TEP_)/(1−f_TEP_) (3)

where Q is the coefficient resulting from the stoichiometry of the titration reaction (Q = m/n, m = number of moles of analyte, n = number of moles of titrant) and M_A_ is the analyte molar mass.

#### Procedure Verification

Instrumental conditions were selected for the developed titration procedure. The procedure was verified by determination of Fe(III) using complexometric titration, in terms of precision, accuracy, and the range of application using synthetic samples and certified reference material. Determination of Fe(III) relied on titration of the sample with EDTA solution in the presence of sulfosalicylic acid used as an indicator [37,38]. Titration was performed in an acidic medium at a pH of about two. In these conditions, a red-purple complex between Fe(III) and sulfosalicylic acid (1:1) was formed in a flask. Then, the sample was introduced into Syringe Pump I and EDTA into Syringe II (Figure 1). The solutions and air met at the confluence point (Figure 1). During the titration process, Syringe Pump I dispensed known ever-decreasing sample volumes (e.g., from 300 to 0 µL, with a 20 μL step) to successive monosegments, while Pump II dispensed ever-increasing titrant volumes (from 0 to 300 μL, with a 20 μL step). Solutions were introduced at the same time (about 13 s) with different flow rates calculated to enable entirely overlapping introduced zones and to facilitate their mixing. Before and after introducing the defined volumes of sample and titrant, a segment of air was inserted to form a monosegment. EDTA mixed with the sample (in the monosegment) replaced sulfosalicylic acid to form a more stable colorless complex with Fe(III) (1:1). Polytetraflouroethylene (PTFE) tubing (ID 0.8 mm) of capacity 1 mL was selected to complete the reaction on the basis of previous experiments [39].

At the moment of the flow-through the flow cell of successive monosegments, analytical signals were recorded and then the solution was directed to waste. After recording all signals, water was used to wash the syringe and the flow cell. The details of the procedure are described in Table 1. The composition of monosegments formed during the titration procedure and signals registered during titration of Fe(III) of concentration 2.00 mg L^−1^ using EDTA of concentration 0.02 mmol L^−1^ as titrant are shown in Figure 2. During the titration the absorbance decreased to a value close to zero, showing that the whole amount of Fe(III) was bound to EDTA (the endpoint of titration). The Fe(III) concentration in the sample was determined on the basis of the EDTA concentration and the dilution factor of EDTA at the endpoint of titration. The way of determining the endpoint of titration and the titrant dilution factor is shown in Figure 3. Fe(II) was determined by applying the same chromogenic reagent, after oxidizing the analyte with the use of H_2_O_2_. The thus prepared sample was subjected to the titration procedure and the sum of Fe(II) and Fe(III) was determined. The Fe(II) concentration was determined from the difference between the sum and the Fe(III) concentration.

Two different total volumes of monosegment (100 and 300 µL) and volume change steps in a monosegment (10 and 20 µL, respectively) were tested, using a titrant of concentration 0.02 mmol L^−1^. A volume of 300 µL and a step of 20 µL were selected because of the possibility of determination of Fe(III) in a wider range of concentrations. Using EDTA at a concentration of 0.02 mmol L^−1^, it was possible to determine Fe(III) in the range 0.35–4.50 mg L^−1^. In selected instrumental conditions, the titration curve was plotted over a period of about 6 min, because of the necessity of refilling the syringes during the titration process. A 2.4 mL volume of sample and the same volume of titrant were consumed for a single titration procedure.

The titration procedure was verified by titration of solutions containing Fe(III) at concentrations (mg L^−1^): 0.50, 1.00, 1.50, 2.00, 3.00, and 4.00 using EDTA solution at a concentration of 0.02 mmol L^−1^. Concentrations of analytes were selected on the basis of similarity to content expected in artesian water samples. Samples were analyzed three times. Calculated values of relative error (RE) and coefficient of variance (CE) for the results were lower than 1.6% and 2.1%, respectively. Therefore, the possibility of determination of Fe(III) in the presence of Fe(II) was studied. To this end, Fe(III) at a concentration of 1.50 mg L^−1^ was determined in the presence of Fe(II) at a concentration (mg L^−1^) of: 0.50, 1.00, 1.50, and 2.00. As the RE for the results was lower than 2.2%, it was assumed that Fe(II) did not influence Fe(III) determination. Finally, the procedure was verified by the determination of Fe(III), the sum of Fe(II) and Fe(III), and, consequently, the content of Fe(II) in the sample. The results of titration are presented in Table 2.

Using the developed procedure, Fe(III) was determined with RE lower than 3.3%. The accuracy of determination of Fe(II) (by difference) was also very good (|RE| < 2.3%). The precision of the procedure was also studied by determination of Fe(III) and Fe(II) in synthetic samples containing Fe(III)/Fe(II) at concentrations (mg L^−1^): 0.50/0.50, 1.00/2.50, and 2.50/1.00. Each sample was analyzed six times. In all samples, Fe(III) and Fe(II) were determined with precision (CV) better than 1.7% and 3.1%, respectively. Figures of merit of the developed procedure are summarized in Table 3.

### 2.3. Analysis of Real Samples

The developed titration procedure was applied to the determination of Fe(II)/Fe(III) in samples of artesian water. Samples were analyzed three times. The results of the determinations using the developed method and the ICP method with appropriate values of confidence intervals (α = 0.05, *n* = 3) are presented in Table 4. The results of determination of the analytes in wastewater certified reference material (directly and with the addition of Fe(III) at a concentration of 0.50 mg L^−1^) are also shown in the table.

It can be assumed that the results of the determination of iron (presented as the sum of Fe(II) and Fe(III)) are consistent with those obtained with the use of the inductively coupled plasma–optical emission spectrometry (ICP–OES) technique and the certified value. It can be noticed that Fe(II) was determined in the wastewater with higher uncertainty, but it should be borne in mind that Fe(II) was determined at a low concentration by difference; however, the sum of Fe(II) and Fe(III) concentrations agreed with the certified value. In conclusion, the procedure was developed for Fe(III) determination, but it can also be applied to the determination of the sum of Fe(III) and Fe(II), and, consequently, Fe(II) determination.

## 3. Methodology

### 3.1. Reagents and Solutions

A stock standard solution of Fe(III) was prepared by dissolving 7.725 g of NH_4_Fe(SO_4_)_2_ 12 H_2_O (POCH S.A., Gliwice, Poland) in HNO_3_ (0.1 mol L^−1^) and making the solution up to 100.0 mL. A stock standard solution of Fe(II) was prepared daily by dissolving 0.176 g of (NH_4_)_2_Fe(SO_4_)_2_ 6 H_2_O (Chempur, Piekary Śląskie, Poland) in 16 mL of H_2_SO_4_ (1 mol L^−1^) and making it up to 25.0 mL with water. The stock solutions were diluted with water to form solutions of appropriate analyte concentration. A solution of sulfosalicylic acid was prepared by dissolving 0.2 g of C_7_H_6_O_6_ 2H_2_O (Chempur, Poland) in HCl (0.1mol L^−1^) in a 100.0 mL volumetric flask. EDTA solution of concentration 0.01 mol L^−1^ was prepared by dissolving 0.373 g of C_10_H_14_N_2_Na_2_O_8_ 2H_2_O (Chempur, Piekary Śląskie, Poland) in water and making the solution up to 100.0 mL. A more diluted titrant solution was prepared by dilution of the above solution with water. A solution of hydrogen peroxide of concentration 0.0348 mol L^−1^ was prepared by appropriate dilution of H_2_O_2_ solution (35%) (Merck, Darmstadt, Germany) with water. Samples for iron determination were prepared by adding sulfosalicylic acid (4 mL, 0.2%) to the sample in the case of determination of Fe(III) or H_2_O_2_ (2 mL, 0.0348 mol L^−1^) and sulfosalicylic acid (4 mL, 0.2%) in the case of determination of the sum of Fe(II) and Fe(III)), and making the solution up to 100 mL with water. Wastewater certified reference material EnviroMAT Waste Water, High (EU-H-3) Lot Number: SC8301825 (SCP SCIENCE, Quebec Canada) was diluted with water in accordance with the manufacturer’s instructions. Samples of water from local artesian wells were collected and analyzed the same day. Samples were acidified [40] and nitrogen was passed through them for about 40 min in order to remove gas interferents (e.g., O_2_, H_2_S). Moreover, water samples were degassed for 15 min using an ultrasonic bath (Sonic 3, Warsaw, Poland). Reagents of analytical grade were used. Substances for preparation of stock standard solutions were weighed to the nearest 0.0001 g. Deionized water (0.05 µS cm^−1^) obtained from the HLP5sp system (Hydrolab, Straszyn, Poland) was used throughout the study.

### 3.2. Instrumentation

A system (FIAlab^®^ Instruments, Seattle, WA, USA) consisting of three syringe pumps (for introducing the sample, titrant, and air, respectively), each equipped with a 9-position selection valve and 1.0 mL Cavro glass barrel syringe was used for the studies. PTFE tubing (0.8 mm i.d.) was used as tubes. A PTFE reaction coil (ID 0.8 mm) of capacity 1 mL was used for iron determination, and a PTFE reaction coil (ID 0.5 mm) of capacity 160 µL was selected for acids and acidity determination. Signals were measured with the use of the USB 4000 Ocean Optics spectrophotometer (Ocean Optics, Dunedin, FL, USA) equipped with fiber optics cables, a halogen light source HL-2000 (Ocean Optics, Dunedin, FL, USA), and a flow cell of light path length 200 mm. Measurements were performed at wavelength 530 nm for determination of iron in the form of a complex with sulfosalicylic acid with a reference scan at wavelength 700 nm.

The ICP–OES method [41] was used as a reference method. Analyses were performed using a SPECTRO ARCOS SOP spectrometer with radial plasma observation (Spectro Analytical Instruments, Kleve, Germany) at operation conditions recommended by the instrument manufacturer. The emission intensities for iron were measured at 259.562 nm.

## 4. Conclusions

The developed approach to titration using a set of syringe pumps is automatic, simple, and convenient in use. From the analytical point of view, it allows you to obtain results of Fe(III) determination with good precision and accuracy. Titration procedure enables determination of the analyte in a wide concentration range, does not need calibration, lasts about 6 min, and consumes 2.4 mL of sample and titrant solutions. The procedure can fulfill the requirements of green analytical chemistry.

The procedure was developed based on a number of ingenious approaches developed earlier. Compared to other methods, it differs in terms of the kind of flow system used, the simplicity of implementation, calculation of titrant dilution factor, and the system automation. It is also characterized by the facility of application to various samples in a wide range of concentrations. This provides a real opportunity to apply it in routine analyses. Compared to tracer-monitored flow titration, the proposed approach uses a simple, reliable, but more expensive flow system which can be applied to tracer-monitored titration. The cost of the flow system can be reduced if, e.g., three-way solenoid valves are used instead of 9-position selection valves. The system also has the potential to be applied to titrations for which it is difficult to find an appropriate tracer. The procedure was designed for flow systems with spectrophotometric detection. The possibility of adapting it to other types of titration and kinds of detection is going to be studied.

## Figures and Tables

**Table 1:** Titration procedure using the system presented in Figure 1. SV—selection valve, SP—syringe pump.

Step	SV Position			SP Flow Rate, µL s^−1^			Volume, µL			Action
	I	II	III	I	II	III	I	II	III	
1	9	9	9	100	100	100	1000	1000	1000	Aspiration of sample, titrant, and air into syringes
2	1	1	1	0	0	100	0	0	100	Introduction of air into reaction coil
3	1	1	1	100	0	0	300	0	0	Formation of zone I in reaction coil
4	1	1	1	0	0	100	0	0	100	Introduction of air into reaction coil
5	1	1	1	94	10	0	280	20	0	Formation of zone II in reaction coil
6	1	1	1	0	0	100	0	0	100	Introduction of air into reaction coil
7	1	1	1	86	20	0	260	40	0	Formation of zone III in reaction coil
8	1	1	1	0	0	100	0	0	100	Introduction of air into reaction coil
9	9	9	9	100	0	100	840	0	400	Aspiration of sample and air into syringes
10	1	1	1	80	30	0	240	60	0	Formation of zone IV in reaction coil
11	1	1	1	0	0	100	0	0	100	Introduction of air into reaction coil
12	1	1	1	74	40	0	220	80	0	Formation of zone V in reaction coil
13	1	1	1	0	0	100	0	0	100	Introduction of air into reaction coil
14	1	1	1	67	50	0	200	100	0	Formation of zone VI in reaction coil
15	1	1	1	0	0	100	0	0	100	Introduction of air into reaction coil
16	1	1	1	60	60	0	180	120	0	Formation of zone VII in reaction coil
17	1	1	1	0	0	100	0	0	100	Introduction of air into reaction coil
18	1	1	1	54	70	0	160	140	0	Formation of zone VIII in reaction coil
19	1	1	1	0	0	100	0	0	100	Introduction of air into reaction coil
20	9	9	9	100	0	100	560	0	500	Aspiration of sample and air into syringes
21	1	1	1	47	80	0	140	160	0	Formation of zone IX in reaction coil
22	1	1	1	0	0	100	0	0	100	Introduction of air into reaction coil
23	1	1	1	40	90	0	120	180	0	Formation of zone X in reaction coil
24	1	1	1	0	0	100	0	0	100	Introduction of air into reaction coil
25	9	9	9	0	100	100	0	900	200	Aspiration of titrant and air into syringes
26	1	1	1	34	100	0	100	200	0	Formation of zone XI in reaction coil
27	1	1	1	0	0	100	0	0	100	Introduction of air into reaction coil
28	1	1	1	27	100	0	80	220	0	Formation of zone XII in reaction coil
29	1	1	1	0	0	100	0	0	100	Introduction of air into reaction coil
30	1	1	1	20	100	0	60	240	0	Formation of zone XIII in reaction coil
31	1	1	1	0	0	100	0	0	100	Introduction of air into reaction coil
32	1	1	1	14	100	0	40	260	0	Formation of zone XIV in reaction coil
33	1	1	1	0	0	100	0	0	100	Introduction of air into reaction coil
34	9	9	9	0	100	100	0	900	400	Aspiration of titrant and air into syringes
35	1	1	1	7	100	0	20	280	0	Formation of zone XV in reaction coil
36	1	1	1	0	0	100	0	0	100	Introduction of air into reaction coil
37	1	1	1	0	100	0	0	300	0	Formation of zone XVI in reaction coil
38	1	1	1	0	0	100	0	0	300	Introduction of air into mixing coil and transport of zones to detector

**Table 2:** Verification of the developed titration procedure: results of determination of Fe(III), the sum of Fe(III) and Fe(II) and Fe(II) as the difference in synthetic samples, EDTA—0.02 mmol L^−1^, CV—coefficient of variation (*n* = 3), RE—relative error.

No.	Fe(III), mg L^−1^		CV, %	|RE|, %	Fe(III) + Fe(II), mg L^−1^	Fe(II), mg L^−1^		CV, %	|RE|, %
	Expected	Determined			Determined	Expected	Determined		
1	0.50	0.50	1.8	0.9	1.01	0.50	0.51	1.8	2.3
2	0.50	0.51	2.4	1.4	2.53	2.00	2.02	2.3	1.0
3	1.00	0.99	2.9	1.4	1.98	1.00	0.99	3.4	0.5
4	1.00	1.02	0.1	2.3	3.54	2.50	2.51	3.7	0.6
5	1.00	1.03	1.5	3.3	4.10	3.00	3.07	0.7	2.3
6	2.50	2.49	1.1	0.3	3.49	1.00	1.00	2.8	0.2

**Table 3:** Figures of merit of the developed titration procedure for Fe(III) determination.

Parameter	Value
Accuracy, |RE|, %	3.3
Precision, CV, % (*n* = 6)	1.7
Sample consumption, mL	2.4
Titrant consumption, mL	2.4
Time of single titration, min	6

**Table 4:** Results of determination of Fe(II) and Fe(III) in samples of artesian water and wastewater certified reference material (WWater, *WWater—wastewater and wastewater with addition of Fe(III) at a concentration of 0.50 mg L^−1^, respectively, ICP–OES—Inductively Coupled Plasma—Optical Emission Spectrometry method; EDTA: 0.02 mmol L^−1^, confidence interval, *n* = 3, α = 0.05.

Sample	Fe(III), mg L^−1^	Fe(II), mg L^−1^	Fe (Total), mg L^−1^	
			Developed Procedure	^1^ ICP–OES/ ^2^ Certified Value
Water 1	0.63 ± 0.01	0.94 ± 0.04	1.56 ± 0.04	^1^ 1.56 ± 0.01
Water 2	0.35 ± 0.01	2.40 ± 0.04	2.75 ± 0.04	^1^ 2.73 ± 0.04
Water 3	0.57 ± 0.03	0.70 ± 0.03	1.27 ± 0.01	^1^ 1.26 ± 0.02
WWater	0.36 ± 0.04	0.13 ± 0.05	0.49 ± 0.03	^2^ 0.49 ± 0.02
^*^WWater	0.85 ± 0.04	0.13 ± 0.05	0.98 ± 0.04	^2^ 0.99 ± 0.02
